# Morphologic variants of Meibomian glands: age-wise distribution and differences between upper and lower eyelids

**DOI:** 10.3389/fmed.2023.1195568

**Published:** 2023-09-04

**Authors:** Saumya Srivastav, Mohammed Hasnat Ali, Sayan Basu, Swati Singh

**Affiliations:** ^1^Centre for Ocular Regeneration, LV Prasad Eye Institute, Hyderabad, Telangana, India; ^2^Department of Computational Bio-Statistics and Data Sciences, LV Prasad Eye Institute, Hyderabad, Telangana, India; ^3^Indian Health Outcomes, Public Health and Economics Research Center, Hyderabad, India; ^4^Shantilal Shanghvi Cornea Institute, LV Prasad Eye Institute, Hyderabad, Telangana, India; ^5^Ophthalmic Plastic Surgery Services, LV Prasad Eye Institute, Hyderabad, Telangana, India

**Keywords:** meibography, dry eye disease, Meibomian glands, tear break-up time, age

## Abstract

**Purpose:**

To evaluate the distribution of various Meibomian gland morphologies across different age groups in healthy individuals.

**Methods:**

The infrared meibographic morphologies of the Meibomian glands from the upper and lower eyelids of 236 healthy individuals (472 eyes; mean age 38.4 ± 17.5 years; 80 female participants: 156 male participants) were evaluated for their prevalence and differences across six decades of life, from 10 to 80 years. A linear mixed-effects modeling test was performed for statistical analysis.

**Results:**

Of 14,452 glands, 8,830 (61%) glands were located in the upper eyelid. No significant differences in frequency were noted between different age groups for distorted, tortuous, hooked, overlapping, abnormal gap, fluffy areas, dropout (except for 51–60 vs. 10–20 years, *P* = 0.023), and thick and thin morphologies. Short glands were significantly more common in individuals aged over 30 years (*P* = 0.015), whereas moderately short and severely short glands were more common in the upper eyelids of individuals older than 50 years compared to those aged 10–20 years (*P* = 0.035). The frequency of distorted, hooked, tortuous, overlapping, and tadpole-shaped Meibomian glands was significantly higher in the upper eyelids than in the lower eyelids for all age groups. Dropout glands were more common in the lower eyelids of individuals younger than 50 years, but no difference was observed in the upper and lower eyelids of individuals over 50 years. Dropout (*P* = 0.006) and severely short glands (0.026) of the lower eyelid were associated with low non-invasive tear break-up time (NIBUT) values.

**Conclusion:**

Various morphologic characteristics of the Meibomian glands that are considered abnormal can be present in healthy individuals, and only moderate to severely short glands display an increase in abnormal morphologic characteristics of the Meibomian glands with age.

## Introduction

Non-contact infrared imaging of the Meibomian glands was introduced in 2008. Since then, numerous studies have described the changes observed in meibography in patients with dry eye disease (DED) (1–9). These changes are essentially morphologic descriptions of the deviation from more or less vertically running normal glands and can be divided based on variations in the length (short or dropout), width (thick, thin, or overlapping), or shape (tortuous, hooked, or distorted) of the glands. The detection of these morphologic alterations and their significance in the context of Meibomian gland dysfunction (MGD) is central to meibography interpretations. Meibographic abnormalities, such as gland dropout and short glands, have been extensively studied in patients with DED, and they were reported to be associated with severe clinical parameters in obstructive MGD (6, 10, 11). Recently, gland tortuosity has also been reported as an early marker of MGD (12). Not all morphologies deviating from the normal vertical gland (reaching up to the tarsal border) are pathologically abnormal. For example, the tortuous glands did not show any histological difference from normal vertically running glands, and the only morphology that had histological differences was severely short glands (<1/3rd of the tarsal height) (13). Hence, morphologies other than severely short glands could be normally present in an individual without representing a dysfunctioning gland. In one study of 42 DED and 33 non-DED individuals, no meibographic differences were observed in the upper eyelids of the two groups, and only the lower eyelids of individuals with DED had more gland loss than those of healthy individuals (10). The prevalence of different morphologies and their comparison across various age groups is not well-studied. Age-related changes in the Meibomian gland function of healthy individuals have been investigated in only one study, where a single Meibographic morphology, i.e., an area without glands, was studied (1, 4, 8, 14, 15). Thus, it is necessary to know the distribution of other morphologies in normal populations, as well as age-related discrepancies that can be within the normal physiologic limits. Therefore, the current study evaluated the frequency distribution of individual Meibographic morphologies across different age groups within a healthy population and explored potential age-related differences.

## Methods

### Study subjects

This prospective observational study followed the tenets of the Declarations of Helsinki and was approved by the LV Prasad Eye Institute Ethics Committee. For all the subjects, informed consent was obtained and was taken from the next of kin for individuals aged under 18 years. A total of 472 eyes of 236 healthy individuals were enrolled in this prospective study. Healthy individuals were defined as individuals with no ocular complaints, an Ocular Surface Disease Index (OSDI) score of <13, no corneal staining, and normal Schirmer values. All subjects had their routine slit-lamp examination and dry eye workup, which included OSDI, non-invasive tear break-up time (NIBUT, Oculus Keratograph 5M, OCULUS GmbH, Wetzlar, Germany), and tear meniscus height (Oculus Keratograph 5M, OCULUS GmbH, Wetzlar, Germany). The exclusion criteria included any ocular surgeries, lid abnormalities such as ectropion, entropion, or cicatrization, long-term contact lens wear, allergies, systemic or autoimmune disorders, and ocular medication use. Sample size calculations were made based on the possibility assumption of a clinically meaningful difference of 5% between the two groups, keeping α at 0.05 a power of 80%, and σ = SD (7%), ε = 5% (2^*^((1.96+0.84)^∧^2)^*^(0.07^∧^2))/(0.05^∧^2) = 30. The minimum number for each group was 30.

### Assessment of infrared meibography images

**Figure 1:**
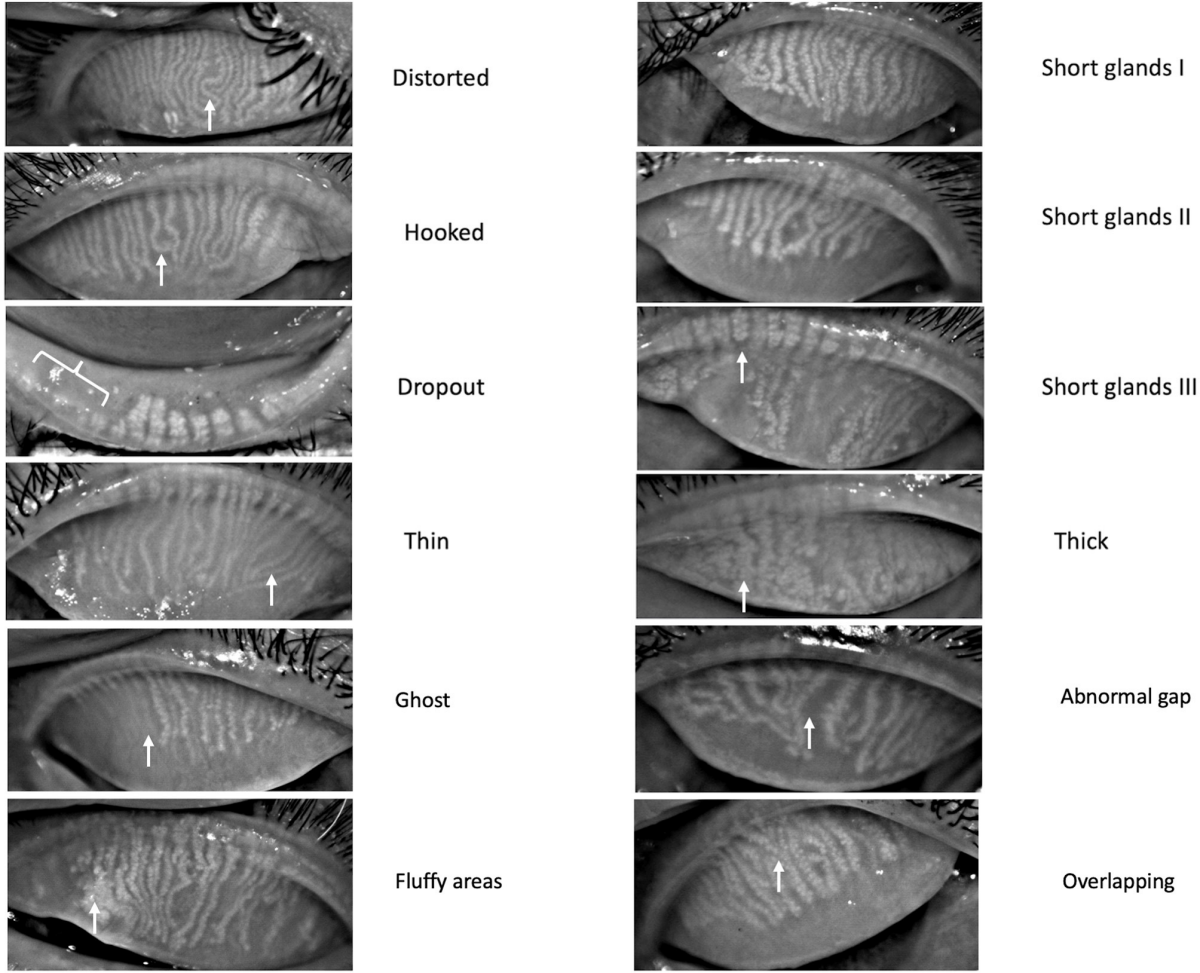
Different Meibomian gland morphologies detected on meibography. White arrows represent the glands involved with the respective morphologic changes.

The definition of dry eye, as outlined in the TFOS DEWS II report, was applied to all healthy individuals following the screening assessment mentioned above. A controlled environment chamber (CAE; with an internal dimension of 6′ × 5′ × 8′, a temperature range of 25 ± 1.0°C, a humidity range of 44 ± 5.0%, and a display LCD of 200 lux) was used to maintain similar environmental conditions. The meibography was assessed by a single observer (S.S.) using the Oculus Keratograph 5M (OCULUS GmbH, Wetzlar, Germany). The infrared meibography/tarsal plate was evaluated as per the description provided by Daniel et al. (5) (Figure 1, distorted: do not follow the parallel way; tortuous: at least one angle of more than 45°; hooked: curled backward looking like a fishhook; dropout: empty space; short grade 1: gland >two-third of the tarsal width; short grade 2: gland extending one-third to two-third of the tarsal width; short grade 3: gland extending < one-third of the tarsal width; thick: gland width twice the normal width; thin: gland width less than half the normal width; overlapping: one gland adjoining the other; ghost: pale and disappearing glands; abnormal gap: spaces abnormally left between the glands; and fluffy areas: white substance like a cluster of mass formation) (5). The above definitions showed good interobserver reliability in the study by Daniel et al. (5). The healthy individuals were divided into six age groups decade-wise (10–20, 21–30, 31–40, 41–50, 51–60, and 61–80 years). Images with less than two-thirds of the eyelid visible on eversion were excluded.

### Statistical analysis

The statistical analysis was performed using linear mixed-effects modeling (R software, Vienna, Austria). The number of glands in each eyelid was counted, and the frequency (%) of each morphology was calculated by dividing it by the total gland count in each eyelid. All the data are presented as mean ± standard deviation (SD). The proportions of different morphologies between the different age groups for each morphology and the differences in the upper and lower eyelids were compared. A *p*-value of < 0.05 was considered significant. Multivariate analysis was performed for assessing the relationship between the NIBUT values (continuous variable) and the presence or absence of various meibographic morphologies.

## Results

**Figure 2:**
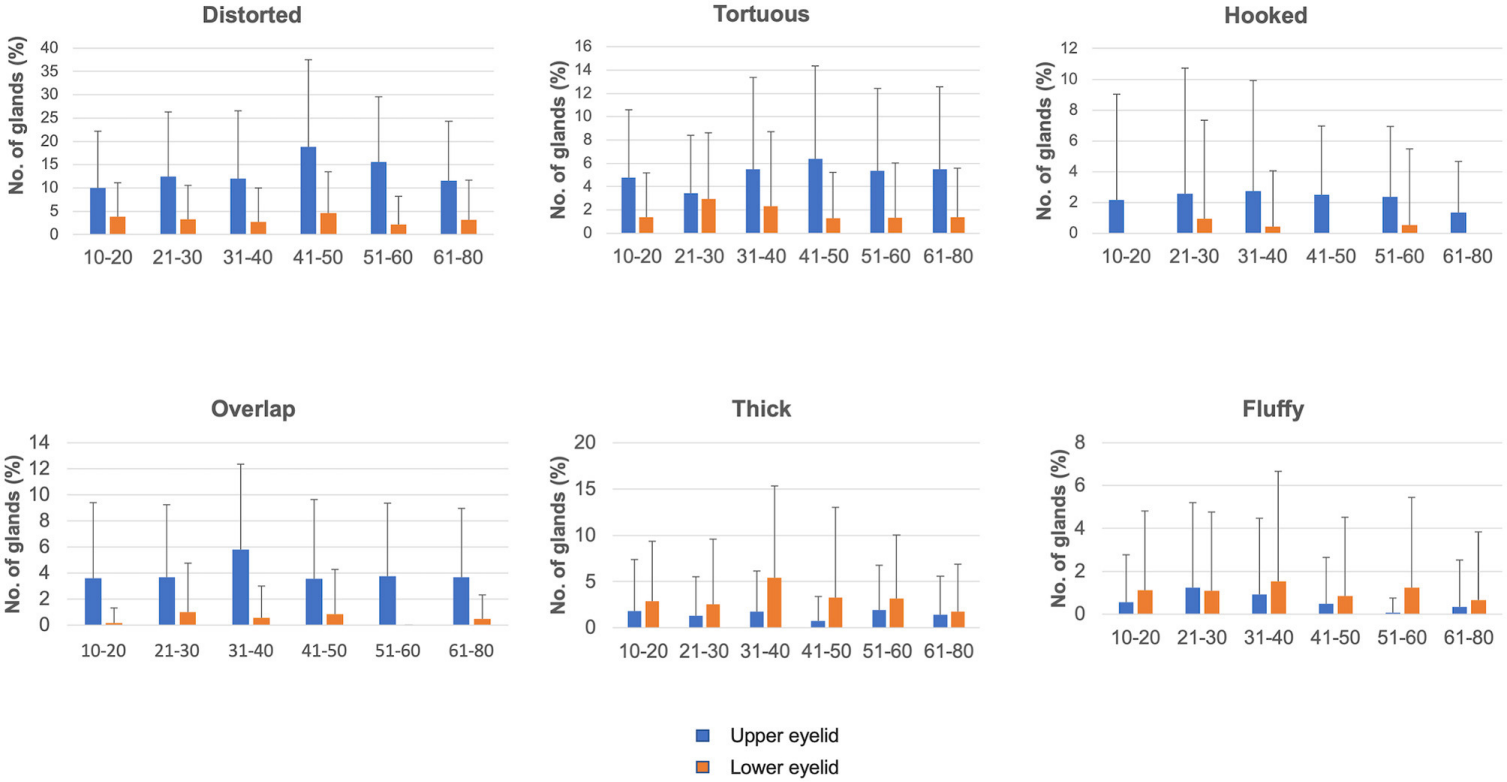
Distribution of different gland morphologies in the upper and lower eyelids across different age groups.

**Figure 3:**
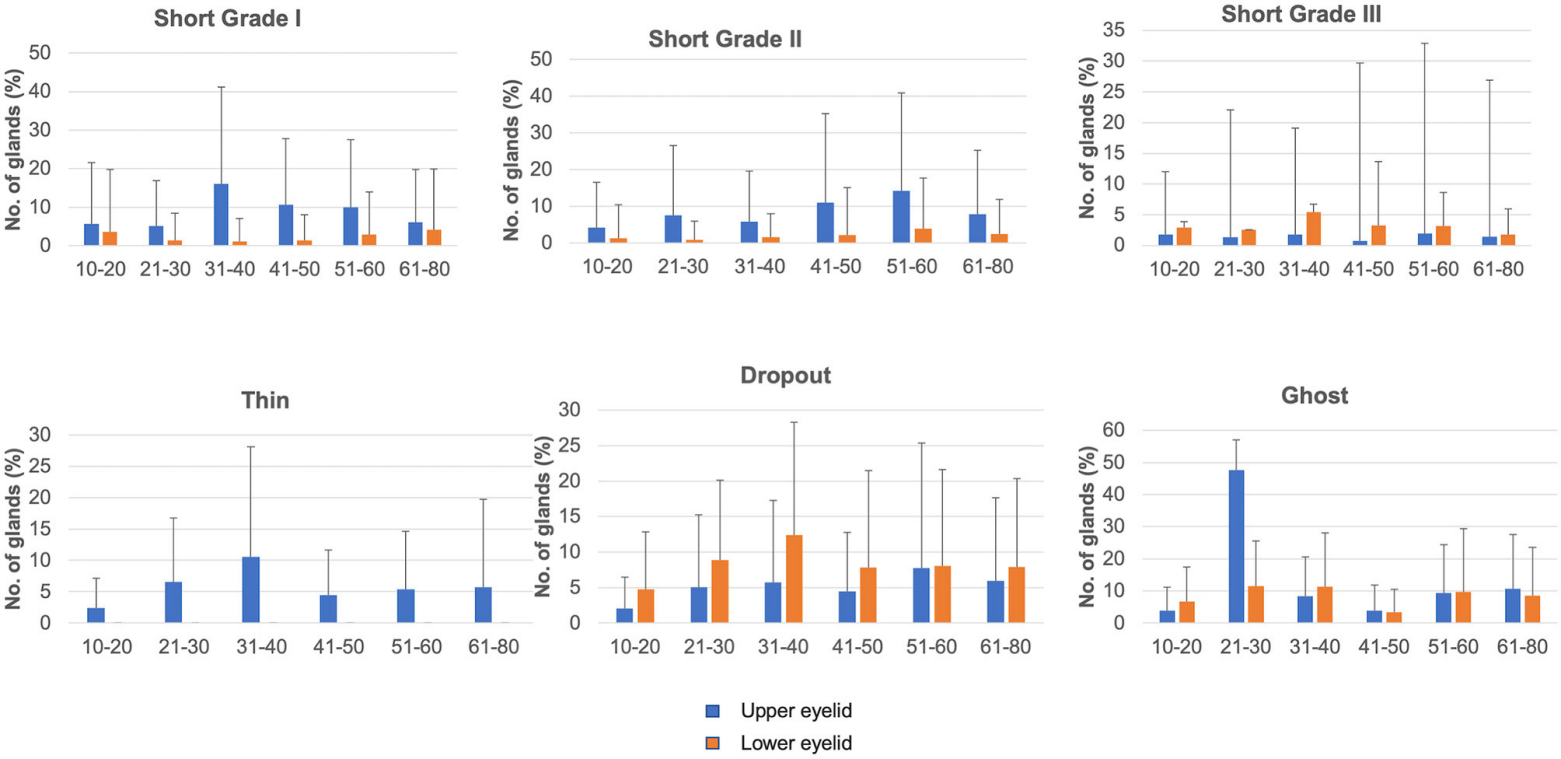
Distribution of different gland morphologies in the upper and lower eyelids across different age groups.

A total of 276 healthy individuals were enrolled. Of these, 236 were confirmed eligible for the study (based on OSDI values and good eyelid eversion), involving 80 female and 156 male participants, with a mean age of 38.41 ± 17.49 years (range 10–80 years; Table 1). The mean OSDI score was 2.3 ± 1.1. In the entire cohort, 9.3% (22/236) had diabetes and 53% (125/236) had refractive errors (presbyopes and myopes). No differences were noted in tear film parameters between emmetropes and individuals with refractive errors (*P* = 0.61). Figures 2, 3 display the frequency distributions of gland morphologies between the upper and lower eyelids.

**Table 1:** Distribution of study subjects and the number of eyelids and glands across different age groups.

	**Total**	**10–20**	**21–30**	**31–40**	**41–50**	**51–60**	**61–80**
Subjects	236	45	46	37	34	42	32
Male:female	156:80	29:16	31:15	29:8	21:13	24:18	22:10
No. of eyelids	944	180	184	148	136	168	128
No. of glands	14,452	3,110	2,955	2,090	1,861	2,437	1,999

The mean frequencies of different morphologies in the upper and lower eyelids are presented in Tables 2, 3. A total of 14,452 glands were evaluated; 8,830 (61.1%) and 5,622 (38.9%) were of the upper and lower eyelids, respectively (Table 1). The total number of glands did not differ between the upper and lower eyelids across different age groups, except for individuals aged < 20 years and those who had more numbers of glands than any other age group (*P* < 0.001; Supplementary material for *P*-values).

**Table 2:** Distribution of different gland morphologies in the upper eyelids of different age groups.

**Meibomian gland morphology**	**% Proportions of each morphology; mean (SD)**					
	**10–20 years**	**21–30 years**	**31–40 years**	**41–50 years**	**51–60 years**	**61–80 years**
Distorted	10 (12.1)	12.5 (13.8)	12 (14.5)	18.8 (18.7)	15.5 (14)	11.5 (12.8)
Tortuous	4.77 (5.8)	3.45 (4.9)	5.50 (7.8)	6.37(7.9)	5.37 (7.0)	5.50 (7.0)
Hooked	2.17 (6.8)	2.55 (8.1)	2.75 (7.1)	2.50 (4.4)	2.36 (4.5)	1.34 (3.3)
Dropout	2.01 (4.4)	5.04 (10.1)	5.73 (11.5)	4.47 (8.3)	7.72 (17.6)	5.98 (11.6)
Short grade 1 (45–50%)	5.73 (15.9)	5.15 (11.7)	16.10 (25.0)	10.69 (17.0)	9.97 (17.5)	6.16 (13.6)
Short grade 2 (55–60%)	4.23 (12.3)	7.63 (18.9)	5.82 (13.7)	11.10 (24.1)	14.30 (26.5)	7.84 (17.4)
Short grade 3 (65–80%)	1.42 (10.2)	10.94 (20.7)	5.31 (17.3)	15.33 (28.9)	15.14 (30.9)	7.91 (25.4)
Thick	1.82 (5.5)	1.31 (4.2)	1.78 (4.3)	0.77 (2.5)	1.93 (4.8)	1.43 (4.1)
Thin	2.42 (4.7)	6.57 (10.1)	10.54 (17.5)	4.48 (7.1)	5.34 (9.2)	5.74 (13.9)
Overlapping	3.60 (5.8)	3.67 (5.5)	5.82 (6.5)	3.57 (6.0)	3.76 (5.6)	3.68 (5.2)
Ghost	3.81 (7.3)	47.65 (9.4)	8.35 (12.2)	3.81 (7.9)	9.25 (15.2)	10.63 (16.9)
Tadpoling	2.19 (4.4)	1.61 (4.0)	1.78 (5.2)	1.98 (7.0)	3.37 (7.8)	2.06 (4.8)
Abnormal gap	2.32 (3.9)	1.47 (3.8)	2.43 (4.5)	1.32 (3.0)	1.58 (3.5)	1.79 (3.7)
Fluffy areas	0.57 (2.2)	1.24 (3.9)	0.93 (3.5)	0.50 (2.1)	0.07 (0.6)	0.34 (2.1)

**Table 3:** Distribution of different gland morphologies in the lower eyelids of different age groups.

**Meibomian gland morphology**	**% Proportions of each morphology; mean (SD)**					
	**10–20 years**	**21–30 years**	**31–40 years**	**41–50 years**	**51–60 years**	**61–80 years**
Distorted	3.8 (7.3)	3.3 (7.3)	2.8 (7.3)	4.6 (8.8)	2.1 (5.9)	3.1 (8.5)
Tortuous	1.37 (3.7)	2.93 (5.7)	2.33 (6.3)	1.28 (3.9)	1.35 (4.6)	1.38 (4.2)
Hooked	0 (0)	0.94 (6.3)	0.42 (3.6)	0 (0)	0.54 (4.9)	0 (0)
Dropout	4.77 (8.0)	8.90 (11.2)	12.39 (15.9)	7.85 (13.6)	8.08 (13.5)	7.91 (12.4)
Short grade 1 (45–50%)	3.61 (16.2)	1.44 (7.0)	1.20 (5.8)	1.47 (6.6)	2.89 (11.0)	4.24 (15.6)
Short grade 2 (55–60%)	1.35 (9.1)	0.86 (5.0)	1.59 (6.4)	2.29 (12.8)	3.94 (13.8)	2.51 (9.4)
Short grade 3 (65–80%)	0.14 (1.0)	0 (0)	0.15 (1.2)	1.26 (10.3)	0.91 (5.5)	0.52 (4.1)
Thick	2.90 (6.4)	2.56 (7.0)	5.41 (9.9)	3.26 (9.7)	3.14 (6.8)	1.77 (5.1)
Thin	0 (0)	0 (0)	0 (0)	0 (0)	0 (0)	0 (0)
Overlapping	0.17 (1.1)	1.01 (3.7)	0.57 (2.4)	0.86 (3.4)	0 (0)	0.47 (1.8)
Ghost	6.66 (10.8)	11.53 (13.9)	11.32 (16.7)	3.28 (7.1)	9.60 (19.8)	8.48 (15.0)
Tadpoling	0 (0)	0 (0)	0 (0)	0 (0)	0 (0)	0 (0)
Abnormal gap	1.71 (4.8)	2.11 (6.0)	1.11 (3.9)	2.21 (8.7)	0.32 (2.9)	0.59 (2.5)
Fluffy areas	1.13 (3.6)	1.09 (3.6)	1.54 (5.1)	0.85 (3.6)	1.24 (4.2)	0.66 (3.1)

### Hooked glands

The distribution of hooked glands was similar across different age groups (mean % ranging from 0 to 2.5; Supplementary material for *P*-values). When compared between the upper and lower eyelids, more hooked glands were present in the upper eyelids across all age groups, except for the 21–30 years age group (Table 4 for *P*-values).

**Table 4:** Differences in the prevalence of various meibographic morphologies between the upper and lower eyelids within specific age groups, along with their significance values.

**Morphologic variants**	**10–20 years**		**21–30 years**		**31–40 years**		**41–50 years**		**51–60**		**61–80**	
	**Mean difference** ^*^	***P*** **-value**	**Mean difference** ^*^	***P*** **-value**	**Mean difference** ^*^	***P*** **-value**	**Mean difference** ^*^	***P*** **-value**	**Mean difference** ^*^	***P*** **-value**	**Mean difference** ^*^	***P*** **-value**
Distorted	0.06	< 0.001	0.09	< 0.001	0.09	< 0.001	0.14	< 0.001	0.13	< 0.001	0.08	< 0.001
Tortuous	0.03	< 0.001	0	0.518	0.03	0.002	0.05	< 0.001	0.04	< 0.001	0.04	< 0.001
Hooked	0.02	0.002	0.02	0.142	0.02	0.005	0.03	< 0.001	0.02	0.011	0.01	0.001
Dropout	−0.03	0.001	−0.04	0.009	−0.07	0.002	−0.04	0.035	0	0.854	−0.02	0.332
Short grade 1 (45–50%)	0.02	0.13	0.04	0.007	0.15	< 0.001	0.09	< 0.001	0.07	< 0.001	0.02	0.456
Short grade 2 (55–60%)	0.03	0.075	0.07	0.001	0.04	0.015	0.09	0.006	0.1	0 < 0.001	0.05	0.011
Short grade 3 (65–80%)	0.01	0.233	0.11	< 0.001	0.05	0.003	0.14	< 0.001	0.14	< 0.001	0.07	0.01
Thick	−0.01	0.16	−0.01	0.182	−0.04	0.001	−0.03	0.027	−0.01	0.127	0	0.636
Thin	0.02	< 0.001	0.07	< 0.001	0.11	< 0.001	0.05	< 0.001	0.05	< 0.001	0.06	< 0.001
Overlapping	0.03	< 0.001	0.03	< 0.001	0.05	0	0.03	0.002	0.04	< 0.001	0.03	< 0.001
Ghost	−0.03	0.029	−0.07	< 0.001	−0.03	0.216	0	0.845	0	0.857	0.02	0.396
Tadpoling	0.02	0	0.02	0	0.02	0.003	0.02	0.022	0.03	0	0.02	0
Abnormal gap	0.01	0.354	−0.01	0.359	0.01	0.052	−0.01	0.371	0.01	0.009	0.01	0.027
Fluffy areas	−0.01	0.203	0	0.816	0	0.461	0	0.404	−0.01	0.012	0	0.492
No extension to the lid margin	0.05	0.066	0.14	0	0.21	0	0.22	0	0.29	0	0.23	0

^*^Mean difference denotes the difference between the upper and lower eyelids of that specific age group; a *P*-value of < 0.05 was considered significant.

### Distorted and tortuous glands

The distribution of the distorted and tortuous glands showed similar frequency across different age groups (mean range, 10–18.8% in the upper eyelids; 2.19–4.64% in the lower eyelids; Supplementary material for *P*-values). The distribution of the distorted and tortuous glands was significantly higher in the upper eyelids than in the lower eyelids across all age groups (Table 4 for *P*-values).

### Short glands and gland dropout

The proportion of dropout glands was similar across different age groups, except for a higher frequency in the upper eyelids of the 51–60 years age group compared to the 10–20 years age group (*P* = 0.023) and in the lower eyelids of the 31–40 years age group compared to the 10–20 years age group (*P* = 0.019). Those under 50 years had more dropout glands in the lower eyelids than in the upper eyelids, whereas no difference between the upper and lower eyelids was noted for those over 50 years of age (Table 3). There were a significant number of upper eyelid short glands in individuals aged over 30 years, with significant differences noted between the following age groups: 31–40 vs. 10–20 years (*P* = 0.015), 31–40 vs. 21–30 years (*P* = 0.007), 61–80 vs. 31–40 years for grade 1 shortening (*P* = 0.064), 51–60 vs. 10–20 years for grade 2 shortening (*P* = 0.044), and 41–50, 51–60 vs. 10–20 years (*P* = 0.035, 0.026) for grade 3 shortening. Lower eyelids exhibited no differences in the prevalence of short glands across different age groups. When compared between eyelids, the upper eyelids of individuals older than 20 years had a significantly greater number of short glands (Table 3).

### Thick and thin glands

The distribution of thick and thin glands was similar across different age groups (Table 2; Supplementary material for *P*-values). More thick glands were present in the upper eyelids than in the lower eyelids of the 31–40 (*P* = 0.001) and 41–50 (*P* = 0.027) years age groups. No differences were noted in the distribution of thin glands between the upper and lower eyelids.

### Other morphologies

No differences were noted in overlapping glands and fluffy areas across different age groups. There were more overlapping glands in the upper eyelids than in the lower eyelids of all age groups (Table 3), whereas fluffy areas were similar in the upper and lower eyelids, except for the 51–60 years age group (*P* = 0.012). Ghost glands were observed more frequently in the 61–80 years age group than in the 10–20 years (*P* = 0.015) and 41–50 years (*P* = 0.024) age groups. No differences were observed in the upper and lower eyelids for ghost glands. No differences were observed in tadpoling and abnormal gap areas across different age groups. More abnormal gap areas were noted in the upper eyelids of individuals over 50 (*P* = 0.009, 0.027), and tadpoling was more prevalent in the upper eyelids across all age groups.

### Relationship with non-invasive tear break-up time

The mean NIBUT was 10.9±2.9 s. After performing multivariate analysis, dropout glands (*P* = 0.006) and severely short glands (*P* = 0.026) of the lower eyelids were associated with lower values of NIBUT.

## Discussion

The current study reports the prevalence of different meibography morphologies in asymptomatic individuals. Different morphologies of the Meibomian glands showed no age-related differences in asymptomatic individuals between 10 and 80 years old, except for short and dropout glands. Meibomian glands undergo shortening and atrophy with age, which can be physiologic and may not always translate into increased OSDI scores.

Meibography studies have focused on the prevalence of the shortening and gland dropouts of the Meibomian gland in DED patients (3–5, 10, 11). The effects of aging on the Meibomian glands have long been studied; however, the prevalence of different morphologies according to age has not been investigated (1–4, 8, 9, 15–17). A cross-sectional study of 236 healthy Japanese individuals revealed an increase in gland dropout areas with age, similar to our study (1). In the current study, gland dropout did not differ across age groups (10–80 years old), except for 51–60 years old vs. 10–20 years old individuals. Gland dropout is the most common morphologic feature that has been studied in the past 40 years. Previously published reports have stated that gland dropout usually begins at 40 years of age (8). Arita et al. suggested that gland dropout begins at the age of 20 in men and at the age of 30 in women (1). From an analysis of our data from when these changes begin to develop, gland dropout was significantly more in individuals aged over 50 years than in the younger group. However, no difference was noted between the 51–60 age group and individuals over 60 years of age. Since our study involved normal subjects, the gland dropout ranged from an overall mean of 5.1–8.2%. In general, more gland dropout has been reported in the lower eyelids (9, 18). We observed more gland dropout in the lower eyelid region of individuals aged < 50 years of age. Similarly, for short glands, a significant increase in prevalence was observed after the age of 30 years in the current study. Earlier studies have implicated the role of androgen in influencing gland shortening after 20 years of age. Crespino et al. did not find any significant difference in various morphologies in the upper eyelids of DED vs. normal individuals; however, the prevalence of only short glands was higher in the lower eyelids of individuals with DED than in those of normal individuals (10). In the current study comprising healthy South Indian eyes, the lower eyelids had no differences in short gland prevalence across different age groups. We also observed no effect of age on non-invasive break-up time in asymptomatic individuals, but dropout and severely short glands were associated with low NIBUT values. Hence, gland shortening in the lower eyelids is likely to represent pathologic changes that might be occurring with age. Histologically, only extremely short glands showed atrophic acini, with decreased PPARY expression and a loss of the Ki-67 expression (13).

Zhao et al. raised the question of whether distorted changes in Meibomian gland morphology are congenital or acquired. More distorted glands have been reported in children with allergic conjunctivitis (2, 3). Gland loss and tortuosity have also been reported in association with long-term contact lens wear (19). In our study, gland distortion was observed in 6.92% of children aged 10–20 years. In healthy asymptomatic individuals, the upper eyelids had more hooked, tortuous, distorted, short, and overlapping glands, whereas the lower eyelids had more gland dropout areas (for < 50 years of age). The distribution of the distorted glands was not affected by age and was observed more in the upper eyelids than in the lower eyelids. The tarsal plate is wider in the upper eyelid than in the lower eyelid, which could be the reason for more tortuous, distorted gland occurrence in the upper eyelids. Histologically, these glands exhibited acinar and ductal histology similar to that of normal glands (12). Recently, tortuous glands were considered an early marker for MGD (5, 12). We found a higher prevalence of tortuous glands in the upper eyelids of asymptomatic individuals, irrespective of the age group. Hence, the correlation of tortuous glands in the causation of MGD should be carefully inspected, which could be a physiologic variation. Pult et al. noted a greater curvature in the Meibomian glands of the upper eyelids and a greater degree of Meibomian gland dropout in the lower eyelids of DED patients (18). However, comparisons between healthy individuals' upper and lower eyelids have not been performed. The clinical relevance of thick and thin glands, overlapping glands, and fluffy regions is not so well-known, although these morphologies were observed in normal individuals in our study. The limitations of our study include the lack of analysis of quality changes in meibum and the inclusion of a subset of the normal population rather than large population-based surveys. Sex-based differences were not analyzed due to the unequal distribution of numbers, with 156 individuals of the male sex. Nonetheless, our recent study showed no sex-based differences in the NIBUT values of healthy individuals (20).

Age-wise prevalence data did not show any difference in the morphologic features of the Meibomian glands in normal individuals with increasing age. Various morphologic characteristics of the Meibomian glands observed in MGD patients can also be present in normal individuals. The relative frequency of abnormal morphologies rather than their absolute presence or absence may be a better indicator of MGD, and this hypothesis needs to be tested in patients with manifest evaporative dry eye.

## Data availability statement

The original contributions presented in the study are included in the article/Supplementary material, further inquiries can be directed to the corresponding author.

## Ethics statement

The studies involving human participants were reviewed and approved by LV Prasad Eye Institute Ethics Committee. Written informed consent to participate in this study was provided by the participants' legal guardian/next of kin.

## Author contributions

SSr, SSi, MH, and SB: concept and design of study or acquisition of data or analysis, interpretation of data, agreement to be accountable for all aspects of the work in ensuring that questions related to the accuracy or integrity of any part of the work are appropriately investigated, and resolved. SSr, SSi, and SB: drafting the article or revising it critically for important intellectual content. All authors: final approval of the version to be published.
