# Supplementary material for: Morphologic variants of Meibomian glands: age-wise distribution and differences between upper and lower eyelids
Source: Front Med (Lausanne). 2023 Sep 4;10:1195568. doi: 10.3389/fmed.2023.1195568 (PMC10507340; doi:10.3389/fmed.2023.1195568)
Supplement: Supplementary file 1 [file Data_Sheet_1.pdf]

Tables demonstrating the differences between various age groups regarding each meibographic morphology mentioned in first cell (G1=10-20; G2=21-30; G3=31-40; G4=41-50; G5=51-60; G6=61-80)

V1	Estimate	lwr	upr	P_values	V12	Estimate3	lwr4	upr5	P_values6	V17	Estimate8	lwr9	upr10	P_values11	V112	Estimate13	lwr14	upr15	P_value
**Distorted_UL_P**					**Distorted_LL_P**					**Tortuous_UL_P**					**Tortuous_LL_P**				
G2 - G1	0.02	-0.05	0.1	1	G2 - G1	0	-0.04	0.03	1	G2 - G1	-0.01	-0.04	0.02	1	G2 - G1	0.02	-0.01	0.04	1
G3 - G1	0.02	-0.06	0.1	1	G3 - G1	-0.01	-0.05	0.03	1	G3 - G1	0.01	-0.02	0.04	1	G3 - G1	0.01	-0.02	0.04	1
G4 - G1	0.09	0.01	0.17	**0.024**	G4 - G1	0.01	-0.03	0.05	1	G4 - G1	0.01	-0.02	0.05	1	G4 - G1	0	-0.03	0.03	1
G5 - G1	0.05	-0.02	0.13	0.72	G5 - G1	-0.02	-0.05	0.02	1	G5 - G1	0	-0.03	0.04	1	G5 - G1	0	-0.03	0.03	1
G6 - G1	0.02	-0.07	0.1	1	G6 - G1	-0.01	-0.05	0.03	1	G6 - G1	0.01	-0.03	0.04	1	G6 - G1	0	-0.03	0.03	1
G3 - G2	-0.01	-0.09	0.07	1	G3 - G2	-0.01	-0.04	0.03	1	G3 - G2	0.02	-0.01	0.06	0.765	G3 - G2	-0.01	-0.03	0.02	1
G4 - G2	0.07	-0.02	0.15	0.313	G4 - G2	0.02	-0.02	0.05	1	G4 - G2	0.03	-0.01	0.06	0.286	G4 - G2	-0.01	-0.04	0.01	1
G5 - G2	0.03	-0.05	0.11	1	G5 - G2	-0.01	-0.05	0.02	1	G5 - G2	0.02	-0.01	0.05	1	G5 - G2	-0.02	-0.04	0.01	1
G6 - G2	-0.01	-0.09	0.07	1	G6 - G2	0	-0.04	0.04	1	G6 - G2	0.02	-0.01	0.06	1	G6 - G2	-0.02	-0.04	0.01	1
G4 - G3	0.07	-0.01	0.16	0.263	G4 - G3	0.02	-0.02	0.06	1	G4 - G3	0.01	-0.03	0.04	1	G4 - G3	-0.01	-0.04	0.02	1
G5 - G3	0.03	-0.05	0.12	1	G5 - G3	-0.01	-0.04	0.03	1	G5 - G3	-0.01	-0.04	0.03	1	G5 - G3	-0.01	-0.04	0.02	1
G6 - G3	0	-0.09	0.08	1	G6 - G3	0	-0.04	0.05	1	G6 - G3	0	-0.04	0.04	1	G6 - G3	-0.01	-0.04	0.02	1
G5 - G4	-0.04	-0.12	0.05	1	G5 - G4	-0.03	-0.07	0.01	0.77	G5 - G4	-0.01	-0.05	0.02	1	G5 - G4	0	-0.03	0.03	1
G6 - G4	-0.08	-0.17	0.01	0.247	G6 - G4	-0.02	-0.06	0.03	1	G6 - G4	-0.01	-0.04	0.03	1	G6 - G4	0	-0.03	0.03	1
G6 - G5	-0.04	-0.12	0.05	1	G6 - G5	0.01	-0.03	0.05	1	G6 - G5	0	-0.03	0.04	1	G6 - G5	0	-0.03	0.03	1

V1	Estimate	lwr	upr	P_values	V1	Estimate	lwr	upr	P_values	V1	Estimate	lwr	upr	P_values	V1	Estimate	lwr	upr	P_value
**Hooked_UL_P**					**Hooked_LL_P**					**Dropout_UL_P**					**Dropout_LL_P**				
G2 - G1	0	-0.02	0.03	1	G2 - G1	0.01	-0.01	0.03	1	G2 - G1	0.03	-0.02	0.08	1	G2 - G1	0.04	-0.02	0.1	0.649
G3 - G1	0.01	-0.02	0.03	1	G3 - G1	0	-0.01	0.02	1	G3 - G1	0.04	-0.02	0.09	0.598	G3 - G1	0.07	0.01	0.13	0.019
G4 - G1	0	-0.03	0.03	1	G4 - G1	0	-0.02	0.02	1	G4 - G1	0.03	-0.03	0.08	1	G4 - G1	0.04	-0.03	0.1	1
G5 - G1	0	-0.03	0.03	1	G5 - G1	0.01	-0.01	0.02	1	G5 - G1	0.06	0.01	0.11	0.023	G5 - G1	0.03	-0.03	0.09	1
G6 - G1	-0.01	-0.04	0.02	1	G6 - G1	0	-0.02	0.02	1	G6 - G1	0.04	-0.02	0.1	0.553	G6 - G1	0.03	-0.03	0.1	1
G3 - G2	0	-0.03	0.03	1	G3 - G2	-0.01	-0.02	0.01	1	G3 - G2	0.01	-0.05	0.06	1	G3 - G2	0.03	-0.03	0.09	1
G4 - G2	0	-0.03	0.03	1	G4 - G2	-0.01	-0.03	0.01	1	G4 - G2	-0.01	-0.06	0.05	1	G4 - G2	-0.01	-0.07	0.06	1
G5 - G2	0	-0.03	0.03	1	G5 - G2	0	-0.02	0.01	1	G5 - G2	0.03	-0.02	0.08	1	G5 - G2	-0.01	-0.07	0.05	1

G6 - G2	-0.01	-0.04	0.02	1	G6 - G2	-0.01	-0.03	0.01	1	G6 - G2	0.01	-0.05	0.07	1	G6 - G2	-0.01	-0.08	0.06	1
G4 - G3	0	-0.03	0.03	1	G4 - G3	0	-0.02	0.01	1	G4 - G3	-0.01	-0.07	0.05	1	G4 - G3	-0.03	-0.1	0.03	1
G5 - G3	0	-0.03	0.02	1	G5 - G3	0	-0.02	0.02	1	G5 - G3	0.02	-0.04	0.07	1	G5 - G3	-0.04	-0.1	0.03	1
G6 - G3	-0.01	-0.05	0.02	1	G6 - G3	0	-0.02	0.01	1	G6 - G3	0	-0.06	0.06	1	G6 - G3	-0.04	-0.11	0.03	1
G5 - G4	0	-0.03	0.03	1	G5 - G4	0.01	-0.01	0.02	1	G5 - G4	0.03	-0.02	0.09	1	G5 - G4	0	-0.07	0.06	1
G6 - G4	-0.01	-0.04	0.02	1	G6 - G4	0	-0.02	0.02	1	G6 - G4	0.02	-0.05	0.08	1	G6 - G4	0	-0.08	0.07	1
G6 - G5	-0.01	-0.04	0.02	1	G6 - G5	-0.01	-0.02	0.01	1	G6 - G5	-0.02	-0.07	0.04	1	G6 - G5	0	-0.07	0.07	1

V1	Estimate	lwr	upr	P_values	V1	Estimate	lwr	upr	P_values	V1	Estimate	lwr	upr	P_values	V1	Estimate	lwr	upr	P_value
**Short_UL_1_P**					**Short_UL_2_P**					**Short_UL_3_P**					**Short_LL_1_P**				
G2 - G1	-0.01	-0.09	0.08	1	G2 - G1	0.03	-0.06	0.13	1	G2 - G1	0.1	-0.02	0.22	0.289	G2 - G1	-0.02	-0.08	0.04	1
G3 - G1	0.1	0.01	0.19	0.015	G3 - G1	0.02	-0.08	0.12	1	G3 - G1	0.04	-0.09	0.17	1	G3 - G1	-0.02	-0.09	0.04	1
G4 - G1	0.06	-0.04	0.15	1	G4 - G1	0.07	-0.04	0.17	0.93	G4 - G1	0.14	0.01	0.28	0.035	G4 - G1	-0.02	-0.09	0.05	1
G5 - G1	0.04	-0.04	0.13	1	G5 - G1	0.1	0	0.2	0.04	G5 - G1	0.14	0.01	0.26	0.026	G5 - G1	-0.01	-0.07	0.05	1
G6 - G1	0	-0.09	0.1	1	G6 - G1	0.04	-0.07	0.14	1	G6 - G1	0.06	-0.07	0.2	1	G6 - G1	0	-0.06	0.07	1
G3 - G2	0.11	0.02	0.19	0.007	G3 - G2	-0.01	-0.11	0.09	1	G3 - G2	-0.06	-0.19	0.07	1	G3 - G2	0	-0.07	0.06	1
G4 - G2	0.06	-0.03	0.15	0.782	G4 - G2	0.04	-0.07	0.14	1	G4 - G2	0.04	-0.09	0.18	1	G4 - G2	0	-0.06	0.07	1
G5 - G2	0.05	-0.04	0.13	1	G5 - G2	0.07	-0.03	0.17	0.55	G5 - G2	0.04	-0.09	0.16	1	G5 - G2	0.01	-0.05	0.08	1
G6 - G2	0.01	-0.08	0.1	1	G6 - G2	0.01	-0.1	0.11	1	G6 - G2	-0.04	-0.17	0.1	1	G6 - G2	0.03	-0.04	0.09	1
G4 - G3	-0.05	-0.14	0.05	1	G4 - G3	0.05	-0.06	0.16	1	G4 - G3	0.1	-0.04	0.24	0.531	G4 - G3	0	-0.06	0.07	1
G5 - G3	-0.06	-0.15	0.03	0.934	G5 - G3	0.08	-0.02	0.18	0.35	G5 - G3	0.1	-0.03	0.23	0.506	G5 - G3	0.02	-0.05	0.08	1
G6 - G3	-0.1	-0.19	0	0.064	G6 - G3	0.02	-0.09	0.13	1	G6 - G3	0.03	-0.12	0.17	1	G6 - G3	0.03	-0.04	0.1	1
G5 - G4	-0.01	-0.1	0.08	1	G5 - G4	0.03	-0.07	0.14	1	G5 - G4	-0.01	-0.14	0.13	1	G5 - G4	0.01	-0.05	0.08	1
G6 - G4	-0.05	-0.15	0.05	1	G6 - G4	-0.03	-0.14	0.08	1	G6 - G4	-0.08	-0.22	0.07	1	G6 - G4	0.02	-0.05	0.09	1
G6 - G5	-0.04	-0.13	0.06	1	G6 - G5	-0.06	-0.17	0.04	1	G6 - G5	-0.07	-0.21	0.06	1	G6 - G5	0.01	-0.06	0.08	1

V1	Estimate	lwr	upr	P_values	V1	Estimate	lwr	upr	P_values	V1	Estimate	lwr	upr	P_values	V1	Estimate	lwr	upr	P_values
**Short_LL_2_P**					**Short_LL_3_P**					**Thick_UL_P**					**Thick_LL_P**				
G2 - G1	0	-0.05	0.04	1	G2 - G1	0	-0.02	0.02	1	G2 - G1	-0.01	-0.02	0.01	1	G2 - G1	-0.01	-0.04	0.03	1
G3 - G1	0	-0.04	0.05	1	G3 - G1	0	-0.02	0.02	1	G3 - G1	0	-0.02	0.02	1	G3 - G1	0.03	-0.01	0.07	0.925
G4 - G1	0.01	-0.04	0.06	1	G4 - G1	0.01	-0.01	0.04	1	G4 - G1	-0.01	-0.03	0.01	1	G4 - G1	0.01	-0.04	0.05	1

G5 - G1	0.03	-0.02	0.07	1	G5 - G1	0.01	-0.01	0.03	1	G5 - G1	0	-0.02	0.02	1	G5 - G1	0	-0.04	0.04	1
G6 - G1	0.01	-0.04	0.06	1	G6 - G1	0	-0.02	0.03	1	G6 - G1	-0.01	-0.03	0.01	1	G6 - G1	-0.01	-0.05	0.03	1
G3 - G2	0.01	-0.04	0.05	1	G3 - G2	0	-0.02	0.02	1	G3 - G2	0.01	-0.01	0.03	1	G3 - G2	0.03	-0.01	0.07	0.381
G4 - G2	0.02	-0.03	0.06	1	G4 - G2	0.01	-0.01	0.04	1	G4 - G2	0	-0.03	0.02	1	G4 - G2	0.01	-0.03	0.05	1
G5 - G2	0.03	-0.01	0.08	0.756	G5 - G2	0.01	-0.01	0.03	1	G5 - G2	0.01	-0.01	0.03	1	G5 - G2	0.01	-0.03	0.05	1
G6 - G2	0.02	-0.03	0.07	1	G6 - G2	0.01	-0.02	0.03	1	G6 - G2	0	-0.02	0.02	1	G6 - G2	-0.01	-0.05	0.04	1
G4 - G3	0.01	-0.04	0.06	1	G4 - G3	0.01	-0.01	0.04	1	G4 - G3	-0.01	-0.03	0.01	1	G4 - G3	-0.02	-0.07	0.02	1
G5 - G3	0.02	-0.02	0.07	1	G5 - G3	0.01	-0.01	0.03	1	G5 - G3	0	-0.02	0.02	1	G5 - G3	-0.02	-0.07	0.02	1
G6 - G3	0.01	-0.04	0.06	1	G6 - G3	0	-0.02	0.03	1	G6 - G3	-0.01	-0.03	0.02	1	G6 - G3	-0.04	-0.08	0.01	0.245
G5 - G4	0.02	-0.03	0.07	1	G5 - G4	0	-0.03	0.02	1	G5 - G4	0.01	-0.01	0.03	1	G5 - G4	0	-0.05	0.04	1
G6 - G4	0	-0.05	0.05	1	G6 - G4	-0.01	-0.03	0.02	1	G6 - G4	0	-0.02	0.03	1	G6 - G4	-0.02	-0.06	0.03	1
G6 - G5	-0.01	-0.06	0.04	1	G6 - G5	0	-0.03	0.02	1	G6 - G5	-0.01	-0.03	0.01	1	G6 - G5	-0.01	-0.06	0.03	1

V1	Estimate	lwr	upr	P_values	V1	Estimate	lwr	upr	P_values	V1	Estimate	lwr	upr	P_values	V1	Estimate	lwr	upr	P_value
**Thin_UL_P**					**Overlapping_UL_P**					**Overlapping_LL_P**					**Ghost_UL_P**				
G2 - G1	0.04	-0.02	0.1	0.758	G2 - G1	0	-0.03	0.03	1	G2 - G1	0.01	0	0.02	0.328	G2 - G1	0.01	-0.05	0.07	1
G3 - G1	0.06	0.02	0.1	0.72	G3 - G1	0.02	0	0.05	0.27	G3 - G1	0	-0.01	0.02	1	G3 - G1	0.05	-0.01	0.11	0.376
G4 - G1	0.02	-0.05	0.09	1	G4 - G1	0	-0.03	0.03	1	G4 - G1	0.01	0	0.02	0.958	G4 - G1	0	-0.06	0.06	1
G5 - G1	0.03	-0.03	0.09	1	G5 - G1	0	-0.03	0.03	1	G5 - G1	0	-0.01	0.01	1	G5 - G1	0.06	0	0.11	0.09
G6 - G1	0.03	-0.03	0.1	1	G6 - G1	0	-0.03	0.03	1	G6 - G1	0	-0.01	0.01	1	G6 - G1	0.07	0.01	0.13	0.015
G3 - G2	0.04	-0.03	0.1	1	G3 - G2	0.02	-0.01	0.05	0.32	G3 - G2	0	-0.02	0.01	1	G3 - G2	0.04	-0.02	0.1	1
G4 - G2	-0.02	-0.09	0.05	1	G4 - G2	0	-0.03	0.03	1	G4 - G2	0	-0.01	0.01	1	G4 - G2	-0.01	-0.07	0.05	1
G5 - G2	-0.01	-0.07	0.05	1	G5 - G2	0	-0.03	0.03	1	G5 - G2	-0.01	-0.02	0	0.102	G5 - G2	0.05	-0.01	0.1	0.337
G6 - G2	-0.01	-0.07	0.06	1	G6 - G2	0	-0.03	0.03	1	G6 - G2	-0.01	-0.02	0.01	1	G6 - G2	0.06	0	0.12	0.064
G4 - G3	-0.06	-0.13	0.01	0.23	G4 - G3	-0.02	-0.05	0.01	0.71	G4 - G3	0	-0.01	0.02	1	G4 - G3	-0.05	-0.11	0.02	0.435
G5 - G3	-0.05	-0.12	0.01	0.414	G5 - G3	-0.02	-0.05	0.01	0.47	G5 - G3	-0.01	-0.02	0.01	1	G5 - G3	0.01	-0.05	0.07	1
G6 - G3	-0.04	-0.12	0.03	1	G6 - G3	-0.02	-0.05	0.01	0.6	G6 - G3	0	-0.01	0.01	1	G6 - G3	0.02	-0.04	0.09	1
G5 - G4	0.01	-0.06	0.08	1	G5 - G4	0	-0.03	0.03	1	G5 - G4	-0.01	-0.02	0	0.376	G5 - G4	0.06	0	0.12	0.125
G6 - G4	0.01	-0.06	0.09	1	G6 - G4	0	-0.03	0.03	1	G6 - G4	0	-0.02	0.01	1	G6 - G4	0.07	0.01	0.14	0.024
G6 - G5	0.01	-0.06	0.07	1	G6 - G5	0	-0.03	0.03	1	G6 - G5	0	-0.01	0.02	1	G6 - G5	0.02	-0.05	0.08	1

V1	Estimate	lwr	upr	P_values	V1	Estimate	lwr	upr	P_values	V1	Estimate	lwr	upr	P_values	V1	Estimate	lwr	upr	P_value
**Ghost_LL_P**					**Tadpoling_UL_P**					**Abnormal.gap_UL_P**					**Abnormal.gap_LL_P**				
G2 - G1	0.05	-0.03	0.12	1	G2 - G1	-0.01	-0.03	0.02	1	G2 - G1	-0.01	-0.03	0.01	1	G2 - G1	0	-0.02	0.03	1
G3 - G1	0.05	-0.03	0.12	1	G3 - G1	0	-0.03	0.02	1	G3 - G1	0	-0.02	0.02	1	G3 - G1	-0.01	-0.03	0.02	1
G4 - G1	-0.03	-0.11	0.05	1	G4 - G1	0	-0.03	0.03	1	G4 - G1	-0.01	-0.03	0.01	1	G4 - G1	0.01	-0.02	0.03	1
G5 - G1	0.03	-0.04	0.1	1	G5 - G1	0.01	-0.01	0.04	1	G5 - G1	-0.01	-0.03	0.01	1	G5 - G1	-0.01	-0.04	0.01	1
G6 - G1	0.02	-0.06	0.1	1	G6 - G1	0	-0.03	0.03	1	G6 - G1	0	-0.02	0.02	1	G6 - G1	-0.01	-0.04	0.01	1
G3 - G2	0	-0.07	0.08	1	G3 - G2	0	-0.03	0.03	1	G3 - G2	0.01	-0.01	0.03	1	G3 - G2	-0.01	-0.03	0.01	1
G4 - G2	-0.08	-0.16	0	0.076	G4 - G2	0	-0.02	0.03	1	G4 - G2	0	-0.02	0.02	1	G4 - G2	0	-0.02	0.03	1
G5 - G2	-0.02	-0.09	0.06	1	G5 - G2	0.02	-0.01	0.04	0.9	G5 - G2	0	-0.02	0.02	1	G5 - G2	-0.02	-0.04	0	0.373
G6 - G2	-0.03	-0.11	0.05	1	G6 - G2	0	-0.02	0.03	1	G6 - G2	0	-0.01	0.02	1	G6 - G2	-0.02	-0.04	0.01	1
G4 - G3	-0.08	-0.16	0	0.099	G4 - G3	0	-0.03	0.03	1	G4 - G3	-0.01	-0.03	0.01	1	G4 - G3	0.01	-0.01	0.04	1
G5 - G3	-0.02	-0.1	0.06	1	G5 - G3	0.02	-0.01	0.04	1	G5 - G3	-0.01	-0.03	0.01	1	G5 - G3	-0.01	-0.03	0.02	1
G6 - G3	-0.03	-0.11	0.05	1	G6 - G3	0	-0.03	0.03	1	G6 - G3	-0.01	-0.03	0.01	1	G6 - G3	-0.01	-0.03	0.02	1
G5 - G4	0.06	-0.02	0.14	0.461	G5 - G4	0.01	-0.02	0.04	1	G5 - G4	0	-0.02	0.02	1	G5 - G4	-0.02	-0.05	0	0.301
G6 - G4	0.05	-0.04	0.13	1	G6 - G4	0	-0.03	0.03	1	G6 - G4	0.01	-0.01	0.03	1	G6 - G4	-0.02	-0.04	0.01	0.88
G6 - G5	-0.01	-0.09	0.07	1	G6 - G5	-0.01	-0.04	0.02	1	G6 - G5	0	-0.02	0.02	1	G6 - G5	0	-0.02	0.03	1

V1	Estimate	lwr	upr	P_values	V1	Estimate	lwr	upr	P_values
**Fluffy.areas_UL_P**					**Fluffy.areas_LL_P**				
G2 - G1	0.01	-0.01	0.02	1	G2 - G1	0	-0.02	0.02	1
G3 - G1	0	-0.01	0.02	1	G3 - G1	0	-0.02	0.02	1
G4 - G1	0	-0.02	0.01	1	G4 - G1	0	-0.02	0.02	1
G5 - G1	-0.01	-0.02	0.01	1	G5 - G1	0	-0.02	0.02	1
G6 - G1	0	-0.02	0.01	1	G6 - G1	0	-0.02	0.01	1
G3 - G2	0	-0.02	0.01	1	G3 - G2	0	-0.02	0.02	1
G4 - G2	-0.01	-0.02	0	0.976	G4 - G2	0	-0.02	0.02	1
G5 - G2	-0.01	-0.02	0	0.094	G5 - G2	0	-0.02	0.02	1
G6 - G2	-0.01	-0.02	0	0.915	G6 - G2	0	-0.02	0.01	1
G4 - G3	-0.01	-0.02	0.01	1	G4 - G3	0	-0.02	0.02	1
G5 - G3	-0.01	-0.02	0	0.696	G5 - G3	0	-0.02	0.02	1
G6 - G3	-0.01	-0.02	0.01	1	G6 - G3	-0.01	-0.02	0.01	1
G5 - G4	0	-0.02	0.01	1	G5 - G4	0	-0.02	0.02	1
G6 - G4	0	-0.01	0.01	1	G6 - G4	0	-0.02	0.02	1
G6 - G5	0	-0.01	0.02	1	G6 - G5	-0.01	-0.02	0.01	1

UL= Upper eyelid

LL=Lower eyelid

Tables demonstrating the differences in each morphology's distribution between upper and lower eyelids of each group

Age_Gr	Types	Diff	Estimate	Lower_CI	Upper_CI	P_Values	Column1	Column2	Column3	Column4	Column5	Column6	Column7
**G1**	Distorted	UL - LL	0.06	0.04	0.09	0							
	Tortuous	UL - LL	0.03	0.02	0.05	0							
	Hooked	UL - LL	0.02	0.01	0.04	0.002							
	Dropout	UL - LL	-0.03	-0.04	-0.01	0.001							
	Short_1	UL - LL	0.02	-0.01	0.05	0.13							
	Short_2	UL - LL	0.03	0	0.06	0.075							
	Short_3	UL - LL	0.01	-0.01	0.03	0.233							
	Thick	UL - LL	-0.01	-0.03	0	0.16							
	Thin	UL - LL	0.02	0.02	0.03	0							
	Overlapping	UL - LL	0.03	0.02	0.05	0							
	Ghost	UL - LL	-0.03	-0.05	0	0.029							
	Tadpoling	UL - LL	0.02	0.01	0.03	0							
	Abnormal.gap	UL - LL	0.01	-0.01	0.02	0.354							
	Fluffy.areas	UL - LL	-0.01	-0.01	0	0.203							

**G2**	Distorted	UL - LL	0.09	0.06	0.12	0
	Tortuous	UL - LL	0	-0.01	0.02	0.518
	Hooked	UL - LL	0.02	-0.01	0.04	0.142
	Dropout	UL - LL	-0.04	-0.07	-0.01	0.009
	Short_1	UL - LL	0.04	0.01	0.06	0.007
	Short_2	UL - LL	0.07	0.03	0.11	0.001
	Short_3	UL - LL	0.11	0.07	0.15	0
	Thick	UL - LL	-0.01	-0.03	0	0.182
	Thin	UL - LL	0.07	0.05	0.09	0
	Overlapping	UL - LL	0.03	0.01	0.04	0

	Ghost	UL - LL	-0.07	-0.1	-0.03	0
	Tadpoling	UL - LL	0.02	0.01	0.02	0
	Abnormal.gap	UL - LL	-0.01	-0.02	0.01	0.359
	Fluffy.areas	UL - LL	0	-0.01	0.01	0.816
**G3**	Distorted	UL - LL	0.09	0.06	0.12	0
	Tortuous	UL - LL	0.03	0.01	0.05	0.002
	Hooked	UL - LL	0.02	0.01	0.04	0.005
	Dropout	UL - LL	-0.07	-0.11	-0.02	0.002
	Short_1	UL - LL	0.15	0.1	0.2	0
	Short_2	UL - LL	0.04	0.01	0.08	0.015
	Short_3	UL - LL	0.05	0.02	0.09	0.003
	Thick	UL - LL	-0.04	-0.06	-0.02	0.001
	Thin	UL - LL	0.11	0.07	0.14	0
	Overlapping	UL - LL	0.05	0.04	0.07	0
	Ghost	UL - LL	-0.03	-0.08	0.02	0.216
	Tadpoling	UL - LL	0.02	0.01	0.03	0.003
	Abnormal.gap	UL - LL	0.01	0	0.03	0.052
	Fluffy.areas	UL - LL	0	-0.02	0.01	0.461
**G4**	Distorted	UL - LL	0.14	0.1	0.19	0
	Tortuous	UL - LL	0.05	0.03	0.07	0
	Hooked	UL - LL	0.03	0.01	0.04	0
	Dropout	UL - LL	-0.04	-0.08	0	0.035
	Short_1	UL - LL	0.09	0.05	0.14	0
	Short_2	UL - LL	0.09	0.03	0.15	0.006
	Short_3	UL - LL	0.14	0.08	0.21	0
	Thick	UL - LL	-0.03	-0.05	0	0.027

	Thin	UL - LL	0.05	0.03	0.06	0
	Overlapping	UL - LL	0.03	0.01	0.04	0.002
	Ghost	UL - LL	0	-0.02	0.03	0.845
	Tadpoling	UL - LL	0.02	0	0.04	0.022
	Abnormal.gap	UL - LL	-0.01	-0.03	0.01	0.371
	Fluffy.areas	UL - LL	0	-0.01	0.01	0.404
**G5**	Distorted	UL - LL	0.13	0.1	0.16	0
	Tortuous	UL - LL	0.04	0.02	0.06	0
	Hooked	UL - LL	0.02	0	0.03	0.011
	Dropout	UL - LL	0	-0.05	0.04	0.854
	Short_1	UL - LL	0.07	0.03	0.11	0
	Short_2	UL - LL	0.1	0.05	0.16	0
	Short_3	UL - LL	0.14	0.08	0.2	0
	Thick	UL - LL	-0.01	-0.03	0	0.127
	Thin	UL - LL	0.05	0.04	0.07	0
	Overlapping	UL - LL	0.04	0.03	0.05	0
	Ghost	UL - LL	0	-0.05	0.04	0.857
	Tadpoling	UL - LL	0.03	0.02	0.05	0
	Abnormal.gap	UL - LL	0.01	0	0.02	0.009
	Fluffy.areas	UL - LL	-0.01	-0.02	0	0.012
**G6**	Distorted	UL - LL	0.08	0.05	0.12	0
	Tortuous	UL - LL	0.04	0.02	0.06	0
	Hooked	UL - LL	0.01	0.01	0.02	0.001
	Dropout	UL - LL	-0.02	-0.06	0.02	0.332
	Short_1	UL - LL	0.02	-0.03	0.07	0.456
	Short_2	UL - LL	0.05	0.01	0.09	0.011
	Short_3	UL - LL	0.07	0.02	0.13	0.01
	Thick	UL - LL	0	-0.02	0.01	0.636

	Thin	UL - LL	0.06	0.03	0.09	0
	Overlapping	UL - LL	0.03	0.02	0.05	0
	Ghost	UL - LL	0.02	-0.03	0.07	0.396
	Tadpoling	UL - LL	0.02	0.01	0.03	0
	Abnormal.gap	UL - LL	0.01	0	0.02	0.027
	Fluffy.areas	UL - LL	0	-0.01	0.01	0.492

Total gland count in upper eyelids of different age groups

TCG_UL

	Estimate	Std. Error	z value	Pr(>|z|)
G2 - G1	-0	0.8	-0	1
G3 - G1	-3	0.8	-4	0
G4 - G1	-3	0.9	-3	0
G5 - G1	-2	0.8	-3	0
G6 - G1	-2	0.9	-2	0.8
G3 - G2	-3	0.8	-3	0
G4 - G2	-2	0.9	-3	0.1
G5 -	-2	0.8	-3	0.1
G6 - G2	-1	0.9	-2	1
G4 - G3	0.3	0.9	0.3	1
G5 - G3	0.5	0.8	0.6	1
G6 - G3	1.2	0.9	1.3	1
G5 - G4	0.2	0.9	0.2	1
G6 - G4	1	0.9	1	1
G6 - G5	0.8	0.9	0.9	1

Total gland count in lower eyelids of different age groups

TCG_LL

	Estimate	Std. Error	z value	Pr(>|z|)	**
G2 - G1	-2	1	-3.8	0	
G3 - G1	-3	1	-5	0	***
G4 - G1	-3	1	-5.5	0	***
G5 - G1	-3	1	-5.1	0	
G6 - G1	-1	1	-2.4	0.3	
G3 - G2	-1	1	-1.4	1	
G4 - G2	-1	1	-2	0.7	
G5 -	-1	1	-1.3	1	
G6 - G2	0.7	1	1.1	1	
G4 - G3	-0	1	-0.6	1	
G5 - G3	0.1	1	0.1	1	
G6 - G3	1.5	1	2.3	0.3	
G5 - G4	0.4	1	0.7	1	.
G6 - G4	1.9	1	2.8	0.1	
G6 - G5	1.4	1	2.3	0.3	
